# Providing wound management in an aged psychiatric facility

**DOI:** 10.1186/1757-1146-4-S1-P55

**Published:** 2011-05-20

**Authors:** Gabrielle Trewin, Gillian Shaw

**Affiliations:** 1 Caulfield Community Health Service. 260 Kooyong rd Caulfield, Victoria, Australia

## 

In January 2010, Podiatry staff at Caulfield Hospital were approached to provide a wound round at Caulfield’s Aged Psychiatric care facility, Namara. Staff at the facility acknowledged an increasing prevalence of foot wounds and a concern was raised by senior staff that the nursing staff had limited wound management knowledge and that it was difficult to transfer the residents to other sites for treatment. The Podiatry department introduced a 3 month Pilot Wound Round in January 2010, involving two Podiatrists and nursing staff from the facility to manage all foot wounds. It was imperative a member of the nursing staff was available at all times to assist with difficult residents. This also provided an opportunity for them to be educated about appropriate wound management skills; including choice of appropriate wound dressings, identifying infection and pressure care. The goals of podiatry intervention were to: (i) identify all foot wounds via referral and manage them on site on a weekly basis to maintain continuity and reduce healing times, (ii) provide treatment on site (nursing staff must accompany residents for any off-site appointments), (iii) educate nursing staff to identify pressure areas and make appropriate referrals, (iv) educate staff about the management of the high risk foot including wound management, pressure care and dressings, (v) improve management of foot complications, (vi) advocate for better access to dressings and equipment for the residents. At the commencement of the round seven wounds were present amongst the 30 residents and after 3 months this number was reduced to 3. Significant improvements were seen with the use of appropriate off-loading care such as felt padding, dressing choice, early referral and strategies to aid prevention. There was also a high focus on continuity of dressing regimes which we audited on a regular basis. Towards the end of the three month pilot, all dressing regimes put in place were being followed appropriately. Limitations to outcomes of the round to date have included the staffs’ knowledge in wound management practice. Whilst one staff member was nominated to attend the wound round with us on a weekly basis, other staff members who attend to dressing changes during the week have not benefited from the Podiatrists expertise. Other barriers have included learning to deal with the often difficult population group with mental illness. Compliance levels varied significantly and overcoming this was often challenging. In conclusion, the wound round has shown to be a cost effective means of reducing the burden of foot related complications in the Aged Psychiatric sector and this pilot has improved awareness and management in the prevention, early identification and management of foot wounds.

